# Investigations on the Degradation Behavior of Processed FeMnSi-xCu Shape Memory Alloys

**DOI:** 10.3390/nano14040330

**Published:** 2024-02-07

**Authors:** Ana-Maria Roman, Ramona Cimpoeșu, Bogdan Pricop, Marius Mihai Cazacu, Georgeta Zegan, Bogdan Istrate, Alexandru Cocean, Romeu Chelariu, Mihaela Moscu, Gheorghe Bădărău, Nicanor Cimpoeșu, Mircea Cătălin Ivănescu

**Affiliations:** 1 Faculty of Materials Science and Engineering, “Gheorghe Asachi” Technical University of Iasi, 41 Dimitrie Mangeron Blvd, 700050 Iasi, Romania; ana-maria.roman@academic.tuiasi.ro (A.-M.R.); ramona.cimpoesu@academic.tuiasi.ro (R.C.); bogdan.pricop@academic.tuiasi.ro (B.P.); romeu.chelariu@academic.tuiasi.ro (R.C.); gheorghe.badarau@academic.tuiasi.ro (G.B.); 2 Physics Department, “Gheorghe Asachi” Technical University of Iasi, 59A Dimitrie Mangeron Blvd, 700050 Iasi, Romania; marius-mihai.cazacu@academic.tuiasi.ro; 3 Faculty of Dental Medicine, “Grigore T. Popa” University of Medicine and Pharmacy, 16 University Street, 700115 Iasi, Romania; mihaela.moscu@umfiasi.ro (M.M.); mircea-catalin.ivanescu@d.umfiasi.ro (M.C.I.); 4 Faculty of Mechanical Engineering, “Gheorghe Asachi” Technical University of Iasi, 43 Dimitrie Mangeron Blvd, 700050 Iasi, Romania; bogdan.istrate@academic.tuiasi.ro; 5 Atmosphere Optics, Spectroscopy and Laser Laboratory (LOASL), Faculty of Physics, Alexandru Ioan Cuza University, 11 Carol I Blvd, 700506 Iasi, Romania; alexcocean@yahoo.com; 6 Laboratory of Applied Meteorology and Climatology, A Building, Physics, Research Center with Integrated Techniques for Atmospheric Aerosol Investigation in Romania (RECENT AIR), Alexandru Ioan Cuza University of Iasi, 11 Carol I, 700506 Iasi, Romania

**Keywords:** biodegradable FeMnSi, in vitro immersion, degradation rate, nano-FTIR

## Abstract

A new functional Fe-30Mn-5Si-xCu (x = 1.5 and 2 wt%) biomaterial was obtained from the levitation induction melting process and evaluated as a biodegradable material. The degradation characteristics were assessed in vitro using immersion tests in simulated body fluid (SBF) at 37 ± 1 °C, evaluating mass loss, pH variation that occurred in the solution, open circuit potential (OCP), linear and cyclic potentiometry (LP and CP), scanning electron microscopy (SEM), energy dispersive spectroscopy (EDS) and nano-FTIR. To obtain plates as samples, the cast materials were thermo-mechanically processed by hot rolling. Dynamic mechanical analysis (DMA) was employed to evaluate the thermal properties of the smart material. Atomic force microscopy (AFM) was used to show the nanometric and microstructural changes during the hot rolling process and DMA solicitations. The type of corrosion identified was generalized corrosion, and over the first 3–5 days, an increase in mass was observed, caused by the compounds formed at the metal–solution interface. The formed compounds were identified mainly as oxides that passed into the immersion liquid. The degradation rate (DR) was obtained as a function of mass loss, sample surface area and immersion duration. The dynamic mechanical behavior and dimensions of the sample were evaluated after 14 days of immersion. The nanocompounds found on the surface after atmospheric corrosion and immersion in SBF were investigated with the Neaspec system using the nano-FTIR technique.

## 1. Introduction

Biodegradable alloys for temporary implants are a special category of materials studied in the scientific world for various medical applications where their presence is necessary to heal a diseased tissue, and later to degrade in the body [1,2]. Compared to conventional permanent implants, this type of biodegradable implant is designed to eliminate possible long-term complications as well as additional surgery. Until healing is complete, the first stage of the reconstruction of the diseased tissue is required, while the implant must maintain its mechanical integrity and then gradually degrade until complete removal [3,4]. Researchers addressing this topic are studying three major classes of Mg, Zn and Fe-based alloys for microsurgical, cardiovascular and orthopedic applications. Biodegradable iron-based materials are considered a suitable solution for medium-time implantation materials, which degrade in the body after a certain healing stage. Manganese alloying with low percentages of silicon can functionalize the alloy to exhibit a shape memory effect (SME) and copper can modify the transformation temperature range and improve the antiseptic property. This alloy belongs to the biodegradable materials class and can be functionalized considering SME for specific medical applications. Each of these classes of biodegradable alloys has advantages and disadvantages in terms of DR, mechanical properties and SME [5]. Mg-based alloys show both biocompatibility and good mechanical properties [6,7,8,9,10,11], but the DR is very high and hydrogen gas evolution has been observed in degradation [12]. Zn shows good biocompatibility, has an intermediate DR between Mg and Fe, and its melting point is low [13]; however, its mechanical properties limit its application in some medical implants [14]. Fe has been a good candidate in biodegradability studies due to its excellent formability and ductility properties, giving the opportunity to realize structures with very fine details, e.g., stents for cardiovascular applications [15]. Another attractive aspect that Fe alloys possess is their biocompatibility and mechanical properties that can be exploited [16,17,18].

The study of pure Fe as a biodegradable alloy has brought to light a very low DR [19], which requires improvements to reach a compromise between the curing period and the maintenance of mechanical integrity for support [20]. Alloying with Mn led to the non-magnetic properties, useful for MRI compatibility, but also for increasing the DR by increasing the Mn content [21,22]. Subsequently, numerous alloying techniques for Fe-Mn with elements such as Si [23], C [24,25,26], Pd [27,28] and Ag [29,30] have been addressed for accelerating the DR, for antimicrobial properties or for obtaining a new bifunctional shape memory alloy (SMA) [31]. A biodegradable alloy with SME and superelasticity would represent a big breakthrough for medical applications of implants due to its healing efficiency [32] by providing real-time adjusted correction or support in minimally invasive surgery implants [33] and crush-resistant stents [34]. Si was a contributory element in the creation of a novel biodegradable and biocompatible alloy that also offers shape memory properties [35]. In vitro cytotoxicity experiments by Liu et al. [36] confirmed the good biocompatibility of Fe--Mn-Si as an SMA for potential stent use. Due to a modification in phase composition caused by increasing Mn concentration, Drevet et al. [37] discovered that the DRs of Fe-Mn-Si SMAs improved with higher Mn concentration, i.e., 0.48, 0.59, and 0.80 mm·y^−1^ for Fe23Mn5Si, Fe26Mn5Si, and Fe30Mn5Si alloys, respectively (Table 1). Cu is an element with known antibacterial properties, combatting certain types of highly resistant bacteria [38]. Biodegradable Fe-Mn-based alloys with Cu addition are less studied in the scientific world, although the data provided so far show that they deserve more attention in further research.

Mandal et al. [39] investigated Fe-Mn-Cu alloys with up to 10%wt Cu added as an antibacterial element. They observed that Fe-25Mn-10Cu has a DR about six times higher than Fe-35Mn (Table 1). The addition of Cu led to excellent cell viability, with a bactericidal effect that recommends it for potential use in internal fracture fixation implants [39]. Ma et al. [40] investigated Fe-30Mn-1C-0.8Cu wt% alloy as a possible material for urinary implants and obtained excellent results showing that the alloy is non-magnetic, biodegradable and has antibacterial and anti-encrustation properties due to its continuous release of Cu ions, making it a good candidate for urinary tract infections and stone treatments. Wei et al. [41] reported the influence of different percentages of Cu (0.5, 1, 1.5 wt%) added to the Fe-30Mn-1C system and found that the DR increased with the increase in Cu wt% associated with the aging treatment (Table 1). Goudarzi et al. [42] investigated the Fe-35Mn alloy with different contents from 0 to 10 wt% Cu and concluded that the compressive strength increased continuously with the increase in Cu content, and reached the maximum value of 203 MPa in the FeMn-10Cu alloy; the average DR was 0.27 mm/year for the FeMn-6Cu alloy, being five times higher than the base Fe-Mn alloy (Table 1); the improvement of magnetic compatibility with MRI was also observed with the increased Cu content, leading to the stabilization of the austenitic phase. Different reported DRs obtained in the corrosion resistance study of biodegradable Fe and FeMn-based alloys in physiological solutions are shown in Table 1.

**Table 1:** Reported results of Fe- and FeMn-based biodegradable alloys according to corrosion resistance tests.

Tested Material	Solution Used in the Experiment	DR		Reference
		Electrochemical mm/Year	Immersion mm/Year	
Pure Fe	Hank’s solution	0.105	-	[43]
Pure Fe	Hank’s solution	0.16	-	[44]
Pure Fe	SBF	0.041	0.101 (70 days)	[45]
Fe-30Mn		0.899	0.028 (70 days)	
Fe-30Mn	SBF	0.29	0.24 (21 days)	[46]
Fe-2Pd	SBF	-	0.025 (70 days)	[47]
Fe-23Mn-5Si	Hank’s solution	0.48	-	[37]
Fe-26Mn-5Si		0.59		
Fe-30Mn-5Si		0.80		
Fe-15Mn-3Si	Ringer’s solution	0.133	0.45 (3 days)	[48]
Fe-14Mn-4Si-2Al	Ringer’s solution	0.054	0.13 (7 days)	[5]
Fe-29Mn-5Si-1Ag	Ringer’s solution	-	0.08 (14 days)	[49]
Fe-29Mn-5Si-2Ag			0.14 (14 days)	
Fe-35Mn-0Cu	Hank’s solution	0.043	-	[39]
Fe-34Mn-1Cu		0.032		
Fe-25Mn-10Cu		0.258		
Fe-30Mn-1C-0.8Cu	Artificial urine solution	-	0.4 (7 days)	[40]
			0.3 (90 days)	
Fe-30Mn-1C-0.5Cu	SBF	0.04	-	[41]
Fe-30Mn-1C-1Cu		0.05		
Fe-30Mn-1C-1.5Cu		0.05		
Fe-35Mn	Hank’s solution	0.05	-	[42]
FeMn-1Cu		0.06		
FeMn-3Cu		0.07		
FeMn-6Cu		0.27		
FeMn-10Cu		0.06		

In this paper, the authors propose a biodegradable material with a possible SME, FeMnSi alloyed with Cu, for implantable medical applications, and discuss the experimental results obtained from chemical, microstructural and phasic analyses, chemical and electrochemical corrosion resistance, dynamic stress behavior and the identification of oxides on the alloy surfaces after different immersion intervals by EDS, FTIR and nano-FTIR analysis.

## 2. Materials and Methods

By melting and remelting the first ingots in a magnetic levitation induction furnace with a cold crucible made by Fives Celes (Lautenbach, France), the Fe29Mn5Si1.5Cu and Fe29Mn5Si2Cu (wt%) SMAs were developed from high-purity materials (99.98% electrolytic Fe, 99.7% Mn, 99.5% Si and 99.9% electrolytic Cu). FeMnSi-1.5Cu and FeMnSi-2Cu were used as further identifiers for the experimental alloys. The final ingots had a diameter of 18.5 mm and a height of 35–40 mm.

Parallelepipedal samples with dimensions of 25 mm × 15 mm × 3 mm and cylindrical samples (used as cast samples in the experiments) with a thickness of 3 mm to 4 mm and 16–18 mm diameter were obtained. The parallelepipedal specimens underwent three rounds of hot rolling at 1050 °C to achieve a thickness of 1 mm.

X-ray diffraction (XRD) studies were used for phase analysis with Expert PRO-MPD equipment (Panalytical type, Cu anode, K-beta (Å) = 1.3923; step scan (2θ): 0.01313; time count for step: 51 (s)). Samples (cast 3 mm × 18 mm; HR 16 mm × 10 mm × 1 mm) were sanded on SiC sheets up to 5000 grit, and rinsed in alcohol for 60 min with ultrasound (PRO 50 ASonic, Ultrasonic Cleaner, Beijing, China).

Hot-rolled (HR) samples cut with an electro-discharge machine (EDM) of 25 mm × 4 mm × 1 mm were tested for DMA, prepared as described in the previous paragraph. The FeMnSi-(1.5-2)Cu HR samples were each subjected to a heating cycle from room temperature, i.e., 25 °C, to 100 °C, at a rate of 3 °C/min. During this time, the samples were deformed by 20 µm, applying a maximum deformation force of 12 N, of which 9 N corresponds to the dynamic part, at a frequency of 1 Hz. The experiments were performed before and after immersing the HR plates for 30 days in SBF at 37 °C. 

The immersion tests were performed using SBF obtained in a laboratory (1000 mL capacity glass beaker (using a hot-plate/magnetic stirrer); Teflon-coated magnetic stirrer + 960 mL deionized water + 6.5456 g NaCl, vigorously stirred at RT for 3 min + 2.2682 g NaHCO_3_, vigorously stirred at RT for 3 min + 0.373 g KCl, vigorously stirred at RT for 3 min + 0.1419 g Na_2_HPO_4_ vigorously stirred at RT for 3 min + heating the solution up to 36.5–37 °C + 0.3049 g MgCl_2_ × 6H_2_O vigorously stirred for 3 min + 9 mL of 1 M HCl solution (added slowly with a dropper) vigorously stirred for 3 min + 0.3675 g CaCl_2_ × 2H_2_O vigorously stirred for 3 min + 0.071 g Na_2_SO_4_ vigorously stirred for 3 min + 6.057 g Tris [=(CH_2_OH)_3_CNH_2_]). 

Cast (3–4 mm × 16–18 mm) and HR (16–20 mm × 8 mm × 1 mm) samples were immersed in individual containers using a ratio of 2 mL/cm^2^ and placed in a thermostatically sealed chamber at 37 °C (for 1, 7 and 14 days). The pH of the solution was registered for the first 72 h with a Hanna HI98191 (Darmstadt, Germany) pH-meter.

For each immersion time, the DR based on the mass loss was obtained for all samples. The samples immersed in SBF were weighed using an AS220 Partner analytical balance (RADWAG Balances & Scales, Radom, Poland). The masses acquired after immersion and ultrasonic rinsing were applied to obtain the DR [mm/y] according to ASTM G31-03 using the following equation (w = mass loss [g], A = sample area [cm^2^], t = time [h] and ρ = density [g/cm^3^]) [50]:

(1) $$DR=\frac{8.76\times 10^{4}W}{At\rho }$$

Electrochemical corrosion tests were performed in an OrigaFlex01A workstation with a three-electrode cell, with saturated calomel electrode and platinum wire as the reference and counter electrodes, respectively. FeMnSi–Cu alloys (samples: 1 mm × 10.5 mm) with an exposed surface area of 0.9 cm^2^ were used as the working electrode. SBF was used as the working liquid. At room temperature (23 °C), the samples were evaluated using LP and CP as well as electrochemical impedance spectroscopy (EIS). The OCP values were acquired 30 min before each test. Potentiodynamic scans were conducted at a scan rate of 1 mV/s. Assessments of LP were performed at a voltage of ±250 mV relative to the OCP. EIS was performed from 50 MHz to 103 Hz with an amplitude of ±1 mV. The EIS recording data were fitted to equivalent circuits and analyzed using ZSimpWin 3.20 software. For each alloy, at least three measurements were performed to ensure reproducibility.

A scattering near-field microscope (neaSCOPE, Haar, Germany) equipped with a tunable laser was used to acquire monochromatic nano-IR images. IR radiation was focused on a metalized tip through a parabolic mirror. The near-field optical contact between the tip and material was manipulated using AFM in tapping mode.

AFM was used to assess the surface morphological modification of the samples before and after the DMA testing (HR plates: 25 mm × 4 mm × 1 mm). The surfaces were scanned with a Si tip (PPP CTR10) with the Nanosurf EasyScan II equipment (Liestal, Switzerland).

A VegaTescan LMH II was used to investigate the structure and aspect of the alloys before and after in vitro tests with SEM. An EDS detector (Bruker, Billerica, MA, USA, X-Flash 6–10) was employed to examine the surface after immersion and to evaluate the chemical composition of the compounds.

## 3. Experimental Results 

### 3.1. Structural and Chemical Analysis of the Experimental Alloys

**Figure 1:**
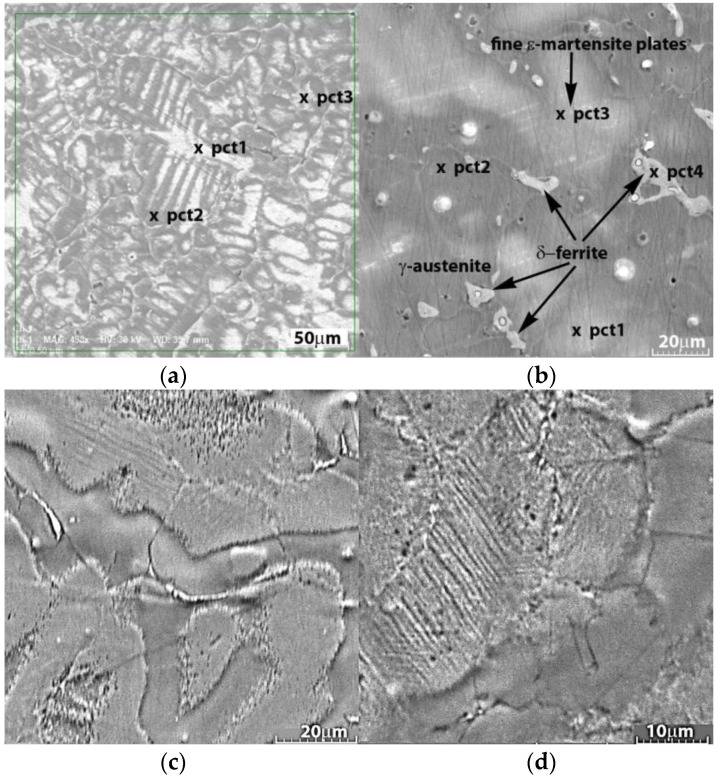
SEM images of the cast alloys’ microstructures: (**a**) FeMnSi-1.5Cu; (**b**) FeMnSi-2Cu and HR; (**c**) FeMnSi-1.5Cu; (**d**) FeMnSi-2Cu.

In the cast state, as shown microscopically in Figure 1a,b, the alloys reveal a dendritic structure with large grains and compounds formed at the boundaries. Figure 1b shows the microstructure of the 2%wt Cu sample in the initial cast state without heat treatment or pre-stressing.

The chemical analysis averages confirm the high quality of the alloy processing technique in levitation furnaces. For the 1.5% Cu alloy (Table 2), lower percentages of Mn, Si and Cu are observed in the solid solution formed, respectively, at point 2, compared to the results obtained on the dendrites appearing in the structure, respectively, at points 1 and 3 (Figure 1a).

On the 2% Cu sample (Table 2), points 1 and 2 are located on the solid solution and point 3 on a zone with martensite plates lower in Mn but also in Si. The δ-ferrite, point 4 in Figure 1b and in Table 2, shows a much higher percentage of Mn compared to the solid solution formed and also higher percentages of Si and Cu. The differences in chemical composition and phases favor the material degradation in smaller pieces [51,52].

**Figure 2:**
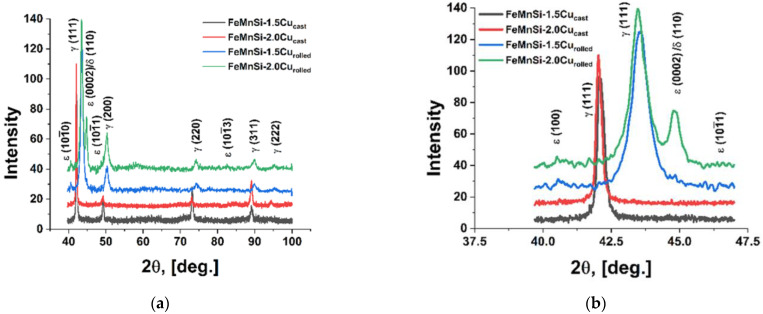
XRD spectra of the alloys: (**a**) FeMnSi-1.5Cu and FeMnSi-2Cu and (**b**) detail 40–47 (2θ).

The experimental materials’ XRD plots (Figure 2) support the findings from the SEM pictures (Figure 1) and show the presence of two phases: γ-austenite as the predominant phase and ε-martensite. 

The effect of Si addition in FeMn alloys was reported by Gavriljuk et al. [53], who showed a lower stacking fault energy of the austenite phase in Fe–Mn–Si alloys with increasing Si content, which also favors the formation of ε-martensite.

### 3.2. Dynamic Mechanical Analysis (DMA)

The behavior of a HR alloy under three-point dynamic stresses was evaluated by a DMA experiment with temperature variation from room temperature (RT) to 100 °C. The stresses occurring in an implant during functioning (during walking for implants in the lower limbs or stretching forces for the upper ones) can be simulated by mechanical–dynamic stress at a certain frequency. 

**Figure 3:**
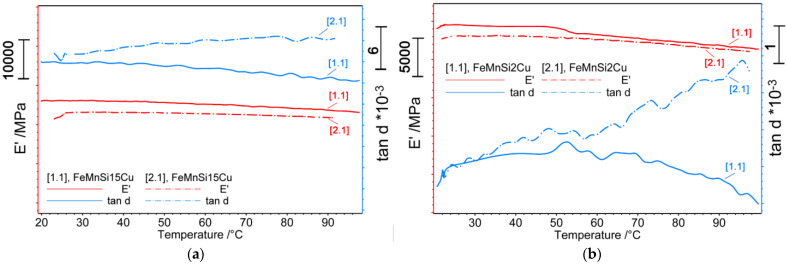
DMA diagrams (heat 25–100 °C, 1 Hz) for the HR plates before (straight line) and after (dashed line) the immersion: (**a**) FeMnSi-1.5Cu (25 × 4 × 1 mm); (**b**) FeMnSi-2Cu (25 × 4 × 1 mm).

The DMA thermograms shown in Figure 3a,b illustrate a continuous decrease in the storage modulus (E′) during heating in all samples because martensite is stiffer than austenite, and two internal friction maxima (tanδ) associated with succeeding recoveries to austenite γ (fcc) of martensite α′-bcc and ε-hcp, respectively [54].

With regard to the FeMnSi-2Cu sample, the thermogram in Figure 3b shows a reduction in the elastic modulus in the temperature domain of 50–60 °C and is associated with the specific solid-state transformations of this alloy [55,56]. 

**Figure 4:**
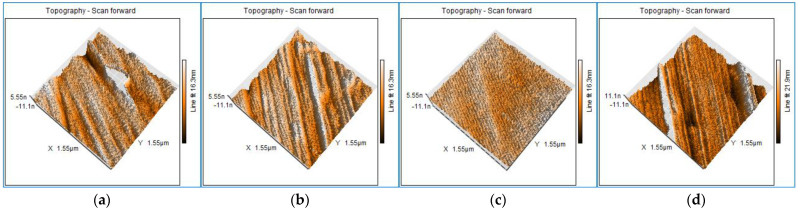
AFM images (3D topography) of the HR plates before DMA testing: (**a**) FeMnSi-1.5Cu; (**b**) FeMnSi-2Cu; and after DMA testing: (**c**) FeMnSi-1.5Cu; (**d**) FeMnSi-2Cu.

Details of the surface profiles in the areas where martensite plates are present are shown in the AFM scans in Figure 4a–d. Sample surfaces were investigated after DMA at a frequency of 1 Hz to observe the profile evolution.

The characteristic plates of the two alloys’ surface profiles, before and after the thermo-dynamic stress, were dimensioned by width (w) and height (h) and exhibited a tendency to decrease in size with mechanical stress (Table 3).

Dimensional measurements were performed using Nanosurf Easyscan 2, version v1-8-0-2 (Liestal, Switzerland) to quantify the impact of the 1 Hz frequency stress on the martensite plates. To observe the deformation tendency, five characteristic groups of martensite plates and five parallel plates within each group were chosen for dimensioning.

The AFM images showed a slight change in the plate dimensions through refinement due to the applied thermomechanical stress. The dimensional change of the plates influences the SME [57,58] and changes the corrosion behavior of the material.

### 3.3. In Vitro Immersion Tests, Degradation Rates and pH Monitoring

The alloy interacts with the liquid medium (in this case, SBF) and releases positive metal ions while retaining electrons on the metal base through the constituent phases, particularly the inter-grain boundary elements. 

During the immersion stage, a protective layer of metal oxide forms on the surface due to chemical interactions. This leads to the passivation of the surface. In the first stage, reactions between the electrolyte solution and the metallic material result in the release of oxides and hydroxides, which modify the pH of the solution. 

**Figure 5:**
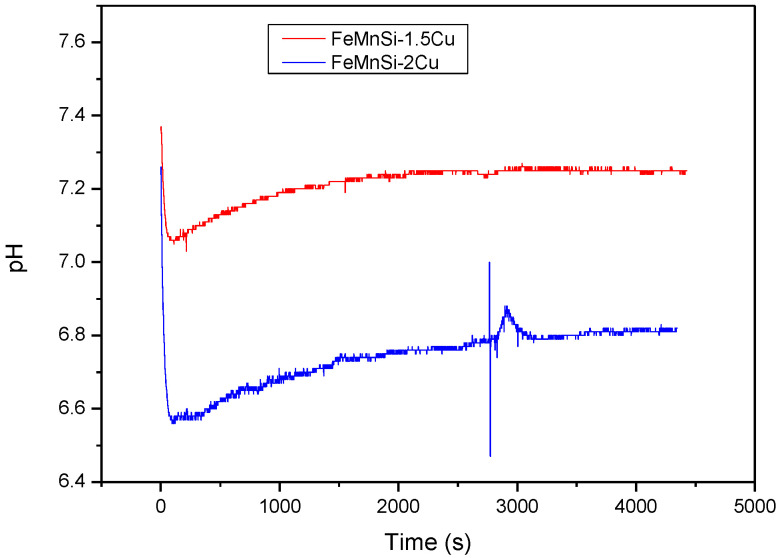
The pH variation recorded during 72 h of immersion in SBF for the HR samples: FeMnSi-1.5Cu; FeMnSi-2Cu.

Two HR FeMnSi-1.5(2)Cu samples were immersed in SBF at 37 °C for 72 h and the pH of the solution was continuously recorded minute by minute. The pH variations during immersion are shown in Figure 5.

When corrosion of iron-based alloys occurs, the DR is driven by the cathodic oxygen reduction reaction [59]:

2H_2_O + O_2_ + 4e^−^ → 4OH^−^, (2)

This reaction was confirmed by the pH variation in both cases (Figure 5). Further, iron hydroxide (Fe(OH)_2_) or hydrating iron oxide (FeO·nH_2_O) are produced when metal ions are released and react with hydroxyl ions [18]:

Fe^+2^ + 2OH^−^ → Fe(OH)_2_, (3)

Some reaction products reached the solution within the first 10 min (600 s) of exposure. The 2% Cu sample shows a significantly faster increase in the solution’s pH than the other alloy, which is likely caused by the generation of a higher number of distinct phases. 

The structural and chemical results obtained after various immersion intervals reveal the behavior of the alloys in SBF and confirm the pH variation due to compound formation over time. For example, after a 14-day immersion, no Ca-based compounds were identified in either sample, and only slight traces of phosphorus were observed on the surface (Table 4). 

The fact that there was an increase in the percentage of Cu following 14 days of immersion, which is primarily due to a decrease in the percentage of Fe cumulated with an increase in the percentage of O on the surface compared to the alloy’s surface after 1 day of immersion, but a decrease in comparison to the amount of O identified after 7 days, indicates that some of the oxides formed between 7 and 14 days have passed into the electrolyte solution. 

According to the mass losses following immersion and using the formula from Equation (1), the material DRs were determined (Table 5).

The addition of copper to FeMnSi alloy has a role in increasing the DR in the SBF environment compared to FeMnSi alloy [42], which encourages the addition of this alloy with a higher number of elements.

The HR alloy, with the exception of the 7-day exposure, shows a higher DR compared to the cast alloy, and this increase may be attributed to the internal stresses accumulated during the rolling process or to the stress-induced martensite formation and local micro-piles, which is also observed and desired for biodegradable Fe. 

For the cast FeMnSi-2%Cu alloy, a rather low DR was obtained over the 1-day immersion, probably as a result of the more resistant passivation layer formed by increasing the percentage of Cu. After its penetration and continued contact with the electrolyte medium, the DR increased considerably, obtaining higher values for the cast alloy compared to the HR one.

Significant differences were obtained between the DRs with increasing Cu percentage from 1.5% to 2%, as confirmed by the structural and chemical aspects. 

**Figure 6:**
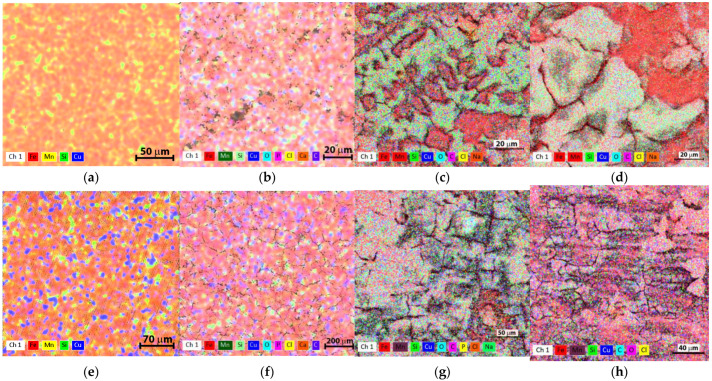
The chemical element distribution of the samples for the initial alloys: (**a**) FeMnSi-1.5Cu, (**e**) FeMnSi-2Cu; after 7 days immersion in SBF, HR: (**b**) FeMnSi-1.5Cu, (**f**) FeMnSi-2Cu; and after 14 days immersion in SBF, cast: (**c**) FeMnSi-1.5Cu, (**g**) FeMnSi-2Cu; HR: (**d**) FeMnSi-1.5Cu, (**h**) FeMnSi-2Cu.

Compared to the results reported by other researchers and presented in Table 1, in the present paper, the authors obtained DRs for the cast samples close to those of the FeMnSi alloy (i.e., 0.45 mm/year for immersion periods of 2 or 4 weeks). The HR samples showed DRs closer to those presented for the FeMn alloys in question around 0.25 mm/year. The distribution of chemical elements (Figure 6) after different immersion intervals indicates generalized corrosion and the growth of a corrosion layer with cracks on the entire surface.

**Figure 7:**
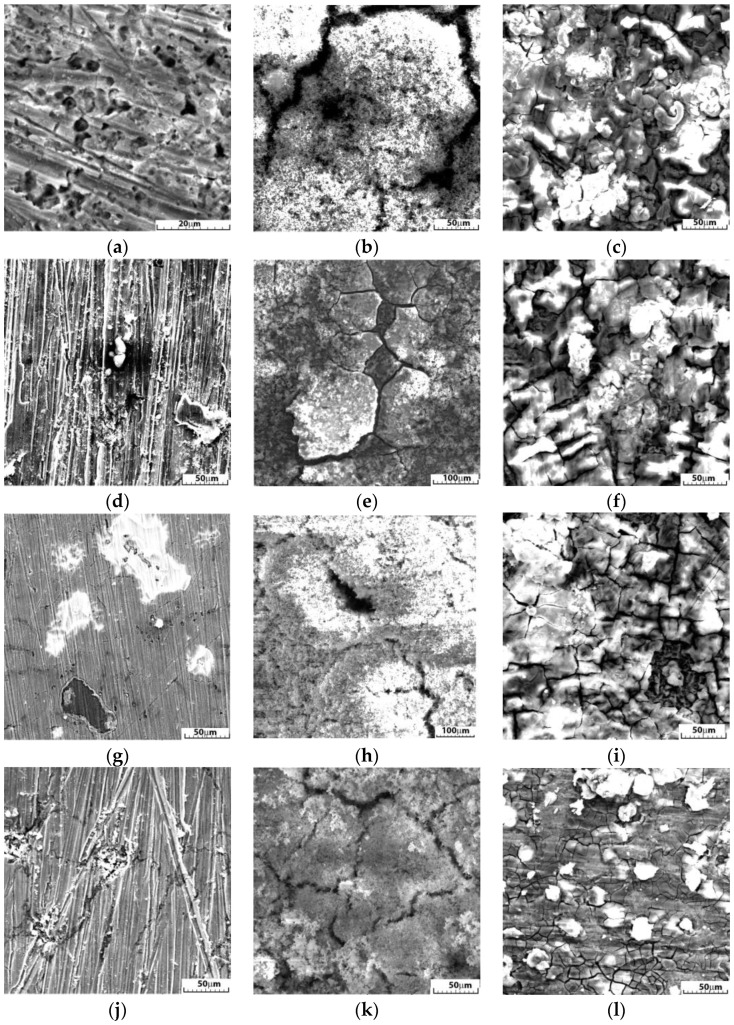
SEM images of the immersed samples in SBF, without DMA testing and immersed for 1, 7 and 14 days in SBF, for the cast alloys: FeMnSi-1.5Cu: (**a**) 1 day, (**b**) 7 days, (**c**) 14 days; FeMnSi-2Cu (**g**) 1 day, (**h**) 7 days, (**i**) 14 days; and the HR alloys: FeMnSi-1.5Cu: (**d**) 1 day, (**e**) 7 days, (**f**) 14 days; FeMnSi-2Cu (**j**) 1 day, (**k**) 7 days, (**l**) 14 days.

The characteristics of the cast and HR samples immersed for 1, 7 and 14 days in SBF were investigated by SEM. All cases showed the formation and intergrowth of an oxide layer and other reaction compounds. After 1 day of immersion, all alloy surfaces showed traces of corrosion, possibly a little more pronounced for cast FeMnSi-1.5Cu, where pitting was noticed (Figure 7a), which could be caused by casting defects.

After 7 days of immersion (Figure 7b,e,h,k), all surfaces showed a discontinuous porous layer of compounds whose intermittency followed the boundary distribution between the grains. After a 14-day immersion, it was noticed that the surfaces were also covered with a more compact layer compared to the samples immersed for 7 days. No differences were observed, at least after 1 day of immersion, between the cast and HR samples, the material condition being an important factor for the first stage of degradation. On the surface, the layer is composed of micrometer-sized components, which are conglomerates of nano-sized particles. This composition can affect degradation and elimination from the body. 

**Figure 8:**
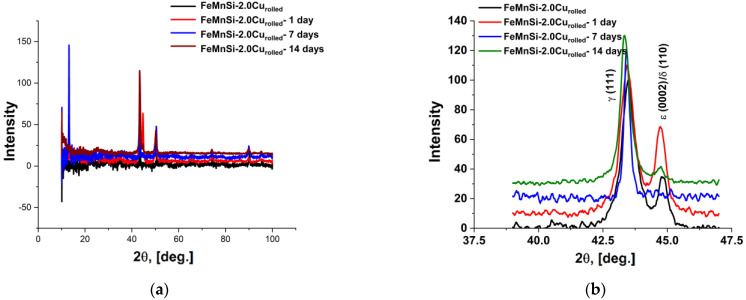
XRD spectra of the alloy after 0, 1, 7 and 14 days of immersion: (**a**) FeMnSi-2Cu and (**b**) detail of the 2θ range: 40–47.5.

The XRD results on the corroded surfaces, presented in Figure 8, after immersion periods of 1, 7 and 14 days, show a reduction in the characteristic peaks of the alloy identified in Figure 2 due to compounds from the corrosion layer that appeared on the surface.

### 3.4. Electrochemical Corrosion Resistance Tests (LP and CP, EIS)

A slight increase in the electrochemical corrosion resistance of the cast alloys was observed with increasing Cu content. The DR of metals is highly dependent on the corrosion potential of the alloy [60]. This potential is influenced by the chemical composition of the alloy and its constituent phases. This can be assessed by connecting the alloy to a standard reference electrode (SRE) and measuring the potential difference. 

**Figure 9:**
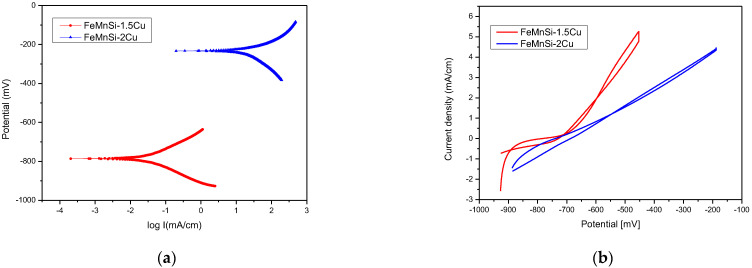
Electro-corrosion results of the experimental FeMnSi-(1.5-2)Cu alloys in SBF: (**a**) Tafel diagrams; (**b**) voltammograms with cyclic curves.

The voltage value and sign are necessary for measuring and reporting corrosion potentials. By increasing the percentage of Cu in the FeMnSi alloy, a decrease in the E_0_ potential was observed. Figure 9a shows a Tafel plot of the linear electrochemical corrosion behavior, which corresponds to a reduction in the corrosion current caused by a slight decrease in the corrosion rate. 

Table 6 shows the indicative corrosion rate results derived from the linear potentiometry data. Even if the anodic and cathodic reactions are closer in intensity, for the FeMnSi–1.5Cu alloy, a reduction in the anode activity was observed by increasing the percentage of Cu to 2%. 

The calculated DRs are closer to those reported in the literature for FeMn-1Cu alloy (approx. 320 µm/year, Table 1) and lower than those presented for alloys with a higher percentage of Cu, e.g., FeMn-10Cu, for which a 10-times-higher DR has been reported, for approx. 250 µm/year where the DR significantly increased due to the different phases that were formed by adding a higher percentage of Cu and accelerating the corrosion through the formation of galvanic micro-piles.

The presence of a hysteresis loop is typically associated with pitting corrosion. The size of the loop is often connected to the number of pits and their depth. Macroscopic and microscopic analysis results (Figure 7) confirm the localized corrosion at a high rate. The corrosion areas were merged, resulting in the transformation of localized corrosion into a more generalized form. Although a similar current density was reached for the two alloys, an increase in the current density from a potential of −700 mV was observed at higher speeds for the 1.5% Cu alloy.

**Figure 10:**
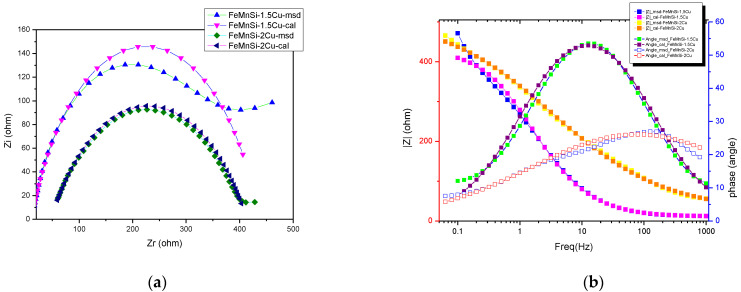
Electrochemical measurements of tested samples: (**a**) Nyquist plots, (**b**) Bode plots.

For each graph in Figure 10a, the Nyguist splitting consists of a semicircle corresponding to the charge transfer reaction (dissolution) of the alloy against the electrolyte solution. These diagrams are not perfect semicircles but are slightly compressed on the imaginary axis. This compression is normally referred to as frequency dispersion and is caused by microscopic and macroscopic surface discontinuities as inhomogeneities [18]. The approximation provided by the recorded data for a semicircle result in a charge transfer resistance (R_ct_) (Table 7), which is expected to be close in value to the R_p_ obtained from LP measurements (Table 6).

EIS was also used to evaluate the electrochemical response of the oxide films formed under exposure to SBF (Bode plots in Figure 10b).

### 3.5. Nano-FTIR Analysis of Corrosion Products

**Figure 11:**
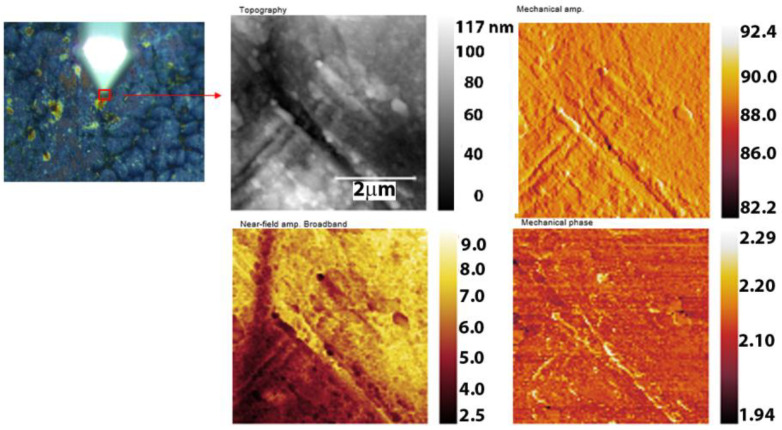
AFM images of the corroded FeMnSi-2Cu sample.

**Figure 12:**
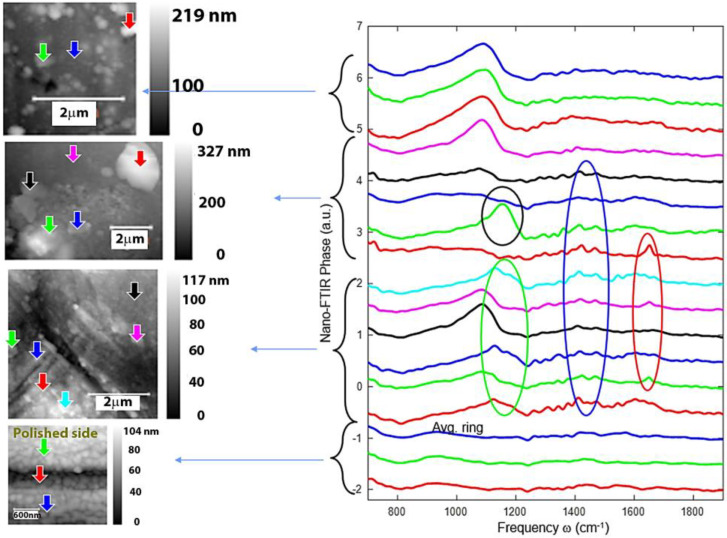
The scanned surface of FeMnSi-2Cu sample and FTIR spectra of the corrosion particles.

Figure 11 and Figure 12 illustrate the oxide compounds discovered on FeMnSi–Cu alloys after 24 h of air contact and immersion in SBF using a new nanoscale analytical method. The nanoscale degradation process was evaluated by analyzing the surface chemicals. During sample scanning, a Michelson interferometer was utilized in the pseudo-heterodyne detection method to identify and analyze the scattered light peak while avoiding far-field background scattering [61]. This allowed simultaneous capture of the AFM topography, mechanical phase, IR absorption parameters and reflectance. The spatial resolution was principally governed by the radius of the probe tip (20 nm), whereas the optical contrast was determined by the local optical properties of the material under the tip and the wavelength of the infrared light. The neaSCOPE system utilizes broadband coherent light in the mid-infrared range from a DFG laser source (Toptica) for nano-FTIR spectroscopy [62].

On the left, Figure 11 displays the scanned surface at 2 μm. The profile and com-pounds observed on the surface were less than 100 nm. Various types of illumination were used to display corroded areas of the surface profile. These include AFM scanning by impinging a laser light focused on a sharp tip of the AFM, and all-optical interferometric scanning which recovers both the amplitude and phase of light scattered from the tip and provides a comprehensive understanding of the complex optical properties of the sample, such as absorption and reflectance.

On the side of interest, there were primarily two types of regions: one with a comparatively thin coating and the other with a pretty thick layer.

Spectra on the polished side were similar to each other, but rather different to the spectra on the polished side of previous samples.

## 4. Discussion

In a major part of the structure, the γ-austenite phase can be seen, and the average grain size can be estimated (from 50 measurements using VegaTc version 3.5.0.0 Measuring feature software) to be about 27 ± 4 µm. Fine plates of ε-martensite can also be observed, which is usually more pronounced in pre-stressed samples. The identification of this phase on unstressed samples is unexpected; however, it has also been observed in other studies, and it is possible that this may result from internal stresses around the nanoparticles found in this type of alloy [63]. The prestressed samples also showed twins generated during mechanical stress (Figure 1c,d). The presence of an additional ferrite (δ-ferrite) phase, located more precisely at the grain boundaries, was observed in the case of the 2%wt Cu alloy and can be attributed to high-temperature residues. 

Depending on the balance between ferrite-stabilizing elements (generally Cr, Si or V, and in this case Si) and austenite-stabilizing elements (Ni, C, Mn), in this case the increase in the percentage of Cu favored the appearance of the δ-ferrite form due to the additional segregation of these elements. It usually occurs in a low volume fraction (less than 2%) [17,64]. An important aspect is that the Mn concentration of 13 to 22 wt% in Fe-Mn binary alloys promotes the formation of ε-martensite phase, whereas a Mn level higher than 22 wt% promotes the γ-austenite formation [49]. Nevertheless, this observation is not as substantial as expected in the case of Fe–Mn–Si alloys because of the addition of 4–5 wt% Si to the alloy. According to these data, the martensitic transformation γ → ε is significantly more visible in Fe26Mn5Si and Fe23Mn5Si alloys than in Fe30Mn5Si alloys. When subjected to external pressures, the elastic influence of the austenitic matrix was identified in alloys with higher Mn concentrations. The mechanical stability of the implant is particularly important for performing its intended function, particularly during the first stage of implantation, when bone remodeling occurs. After the bone remodeling stage, the period of implanted element degradation may begin. Degradation of the metal element must occur homogeneously in smaller pieces and in the shortest possible time. 

To evaluate the mechanical behavior of the experimental alloys investigated in this work, DMA tests were performed before and after a 14-day immersion of the plates in SBF. The viscoelastic properties of the materials as a function of temperature variation, immersion time in SBF, and frequency (1 Hz) did not change for any of the two alloys. However, despite the short immersion period, the specimens showed a high degree of surface corrosion (Figure 6, Figure 7 and Figure 8) which may affect the mechanical behavior of the specimen under external loads. For a more precise assessment of the mechanical property evolution over the entire degradation period, further tests are required. Both alloys show similar tendencies of elastic modulus evolution with temperature. More recent studies have reported cross-correlated variations in the storage modulus and magnetization dung heating. Mocanu et al. [65] observed that the occurrence of the first tanδ maximum around 50 °C could be a composed effect from the superposition of the inverse martensitic ε-(bcc) → γ (fcc) transformation and the antiferromagnetic → paramagnetic transition at Néel temperature (NT) [66]. Further heating of the alloys to the initial state showed a decrease in the tand factor, especially for the 2%wt Cu alloy, associated with the mechanical energy dissipation capacity of the alloy [67] due to the partial transformation of the martensite.

After the immersion process of 14 days, in both cases, the same tendencies for a slight decrease in the elastic modulus with increasing temperature (the dotted lines in Figure 3a,b) and an increase in the dissipation capacity of the alloy with heating were observed. In contrast to the sample containing only 1.5% Cu, increasing the proportion of Cu influenced the change in the elastic modulus over temperature by more martensite transitioning into austenite. 

The behavior of the experimental alloys in SBF was analyzed by immersing the samples at 37 °C and then maintaining them for 1, 7 and 14 days. The samples were flipped from side to side every 12 h for exposure for equal periods of time. For the results of the chemical analysis, the EDS technique was followed.

The metal surfaces were analyzed after the mentioned immersion intervals and are shown in Table 4. The results show surface oxidation after immersion and an increase in the percentage of O with increasing maintenance of the sample in the solution. In addition to oxygen, other chemical elements such as calcium and phosphorus due to natrium phosphate, calcium chloride, or carbon and chlorine were identified.

XRD results from the alloys’ surfaces after immersion periods confirmed the corrosion process starting from day one, after which immersion peaks of FeOOH, MnOOH, FeCO_3_, MnCO_3_ and CaCO_3_ can be observed [68]. The presence of ε-martensite phase (Figure 8b) increases the degradation rate of the material, forming micro-piles between material different phase constituents. 

The polarization resistance (R_p_) measured from the LP experiments was found to be only slightly different for the two alloys. This increase in polarization resistance can be attributed to the appearance of the δ-phase at the grain boundary for the 2%wt Cu sample. The corrosion current density decreases with increasing Cu percentage from 1.5% to 2%, indicating that the passive layer formed by oxidation on the surface is more stable at higher Cu percentages. CP tests (Figure 9b) are commonly used to assess the susceptibility of an alloy to pitting corrosion in an electrolyte solution. The potential was altered in just one cycle, and the size of the hysteresis loop was studied in connection with the values of the OCP and the passivation potential on the return junction, which indicated a generalized corrosion pattern for the two alloys. 

As mentioned in the experimental section, EIS measurements were performed in the passivity region every 50 mV by an anodic potential sweep. In Figure 10, the Bode variations for the two alloys are plotted to illustrate the impedance evolution with increasing frequency. The general pattern is the same for both samples and features an increase in impedance with increasing process frequency, which can be correlated with a gradual thickening of the oxide film from the alloy surface. The obtained spectra were modeled using the equivalent electrical circuit shown in Table 7, which comprises an array with an R_ct_ element. 

A good accordance between the experimental and figured data can be observed from the graph shown in Figure 10b. In this model, R_s_ represents the resistance of the electrolyte solution, and the high-frequency time constant R_ct_-CPE is related to the charge transfer resistance and double-layer capacitance at the oxide–electrolyte layer interface. Given the semiconducting nature of the film formed from Fe and Mn oxides in general and with a slight influence of Si and Cu, the double-layer capacitance should incorporate the contributions of the space charge capacitance, CSC, and the Helmholtz layer capacitance, CH, so that the overall CPE can figure as a combination of these two components in series. For this circuit, a low-frequency time constant can be added that can be correlated with redox processes in the oxide layer. Since the initial increase indicates a reduction of redox activity, probably because as the film thickness increases, it adopts a more corrosion-stable structure, the introduction into the fission circuit was discarded [61]. 

A broad band can be observed around 1100 cm^−1^, which was the strongest at points with less material and also in the thick layer (top spectra). We observed several features; most of them were repeatedly observed in several spectra. Such features are marked roughly with colored circles. Note that not all the spectra within the circles always showed the specific feature. At 1382 and 1637 cm^−1^, the presence of copper oxide can be considered to appear in the oxide layer formed on the surface [62]. Aside from the thick layer spectra, a significant and distinct band at about 1100 cm^−1^ (indicated with a blue circle) was noticed and has three possible hypotheses: the presence of SiO_2_, a carbonate compound or the presence of a C-O-C functional group [69]. 

A spectrum particle with a strong and broad band, shown in Figure 12 (similar to phonon absorption of oxides), was discovered on the thin layer, indicating the presence of a common inorganic ion such as CO_3_^2−^ (results presented in Table 4). Simultaneously, Veneranda et al. found the compound goethite (α-FeOOH) at 798 cm^−1^, which was more likely to cover the entire surface of the material [70,71]. In the thick layer, two particles with a definite peak were detected at roughly 1590 cm^−1^. The spectra are rather noisy because the near-field amplitude of these particles is relatively small. Su et al. discovered the presence of COO^−^ at the 1590 cm^−1^ peak, which can potentially be assigned to the carboxylate group [72,73]. 

## 5. Conclusions

From a general standpoint, adding Cu to FeMnSi alloy is favorable for its antimicrobial effect, as well as for improving the SME, workability and corrosion resistance of these alloys.

Cu addition to FeMnSi alloy can influence the DR in two aspects: it can reduce the corrosion process by contributing to surface stabilization through the copper oxides it forms, or it can increase the DR by separating it at the grain boundaries or in certain phases and intermetallic compounds that contribute to the formation of galvanic microstacks inside the solid solution composed from the main elements, namely Fe, Mn and Si. It is probable that these processes will participate to varying degrees during the duration of the alloy’s contact with the electrolyte solution, depending on which factors are more favorable. No significant changes in mechanical properties were observed after 14 days of immersion. Nano-sized corrosion traces were found on the surface after 24 h of contact with an electrolyte solution. 

## Figures and Tables

**Table 2:** Initial chemical composition of the experimental alloys: (a) FeMnSi-1.5Cu; (b) FeMnSi-2Cu.

(a) FeMnSi-1.5Cu	Fe (wt%)	Mn (wt%)	Si (wt%)	Cu (wt%)
Point 1	56.15	34.03	5.89	1.92
Point 2	65.49	29.46	3.94	1.11
Point 3	55.87	34.04	5.69	1.73
Average	59.17	32.51	5.17	1.59
(b) FeMnSi-2Cu	Fe (wt%)	Mn (wt%)	Si (wt%)	Cu (wt%)
Point 1	55.22	35.91	5.95	2.92
Point 2	60.73	32.08	4.98	2.22
Point 3	66.40	28.41	3.81	1.38
Point 4	41.85	42.54	7.01	8.58
Average	60.78	32.13	4.91	2.17

St.dev.: Fe: ±0.18; Mn: ±0.08; Si: ±0.05; Cu: ±0.05.

**Table 3:** AFM results of measured martensite plates (average of 5 determinations on 5 plates for each sample), before and after DMA testing, for FeMnSi-1.5Cu and FeMnSi-2Cu samples.

Sample	Dimension	Average—Before DMA (nm)	St. Dev. ±	Average—After DMA (nm)	St. Dev. ±
FeMnSi-1.5Cu (1 Hz)	w	368	24.6	92	3.8
	h	234	12.6	46	3.1
FeMnSi-2Cu (1 Hz)	w	200	18.6	163	13.3
	h	116	6.5	71	11.5

**Table 4:** The chemical composition of the 3 alloys FeMnSi-(1.5-2)Cu, cast (C) and HR, after 1, 7 and 14 days (D) of immersion in SBF and after the ultrasonic cleaning (I + U).

D I + U	Alloys/wt%		Fe	Mn	Si	Cu	O	C	Cl	Ca	P
1	FeMnSi-1.5Cu	C	70.7	2.36	4.99	1.53	9.66	10.2	-	-	0.38
		HR	49.8	25.1	4.32	1.52	11.8	5.9	-	0.29	1.27
	FeMnSi-2Cu	C	62.2	15.8	4.11	1.13	10.4	5.23	-	-	0.99
		HR	52.9	24.6	3.73	1.66	9.75	6.44	0.1	-	0.72
7	FeMnSi-1.5Cu	C	31.4	7.8	2.06	2.18	37.5	9.95	0.63	1.64	6.84
		HR	32.1	8.65	0.72	0.49	36.5	8.77	3.48	2.09	7.21
	FeMnSi-2Cu	C	34.3	4.18	1.41	0.13	37.6	11.8	0.8	1.44	8.34
		HR	28.9	7.73	0.41	0.25	40.9	7.93	1.81	2.5	9.54
14	FeMnSi-1.5Cu	C	29.6	13.6	6.03	5.79	34.6	9.44	0.38	-	-
		HR	29.1	14.2	3.74	5.74	37.1	9.73	0.21	-	-
	FeMnSi-2Cu	C	25.6	11.8	3.9	8.8	37.6	10.9	0.46		0.6
		HR	30.9	14.7	3.5	8.59	31.7	9.59	0.87	-	-
EDS detector error %			0.38	0.18	0.07	0.07	2.14	0.77	0.05	0.05	0.19

St.dev.: Fe: ±0.5; Mn: ±0.35; Si: ±0.2; Cu: ±0.2; O: ±1; C: ±0.25; Cl: ±0.1; Ca: ±0.01; P: ±0.01.

**Table 5:** DRs determined by each mass gain/loss of the experimental alloys: (a) FeMnSi-1.5Cu; (b) FeMnSi-2Cu; C and HR, subjected to 1, 7 and 14 days of immersion in SBF and subsequent ultrasonic cleaning.

(a) FeMnSi-1.5Cu	1 day		7 days		14 days	
	C	HR	C	HR	C	HR
Initial mass (mg)	6007.4	698.2	3192.4	851	3471.6	794
Mass after immersion (mg)	6010.2 (+2.8)	697.4 (−0.8)	3153.8 (−38.6)	858.6 (+7.6)	3436.8 (−34.8)	767.6 (−26.4)
Mass after ultrasound (mg)	6005.9 (−1.5)	697.3 (−0.9)	3147.2 (−45.2)	844.8 (−6.2)	3434.4 (−37.2)	766.9 (−27.1)
DR (μm/year)	108	184	536	145	200	327
(b) FeMnSi-2Cu	1 day		7 days		14 days	
	C	HR	C	HR	C	HR
Initial mass (mg)	5628.8	716.8	4886.4	977.1	6122.4	1100.5
Mass after immersion (mg)	5629.9 (+1.1)	716.4 (−0.4)	4855.8 (−30.6)	970.3 (−6.8)	6063.8 (−58.6)	1102.7 (+2.2)
Mass after ultrasound (mg)	5628.6 (−0.2)	716.2 (−0.6)	4850 (−36.4)	966.6 (−10.5)	6058 (−64.4)	1087 (−13.5)
DR (μm/year)	16	134	418	204	369	115

The sample areas: 1.5Cu (1 day) C = 6.8 cm^2^, 1.5Cu (1 day) HR = 2.4 cm^2^, 1.5Cu (7 days) C = 5.9 cm^2^, 1.5Cu (7 days) HR = 3 cm^2^, 1.5Cu (14 days) C = 6.5 cm^2^, 1.5Cu (14 days) HR = 2.9 cm^2^; 2%Cu (1 day) C = 6.1 cm^2^, 2Cu (1 day) HR = 2.2 cm^2^, 2Cu (7 days) C = 6.1 cm^2^, 2Cu (7 days) HR = 3.6 cm^2^, 2Cu (14 days) cast = 6.1 cm^2^, 2Cu (14 days) HR = 4.1 cm^2^.

**Table 6:** Electrochemical corrosion parameters (in SBF) of FeMnSi-Cu alloys.

Sample	E_0_ mV	b_a_ mV	b_c_ mV	R_p_ ohm·cm^2^	J_corr_ mA/cm^2^	V_corr_ μm/Year
FeMnSi-1.5Cu	−786	92	−85	638	32.1	374
FeMnSi-2Cu	−232	68	−124	847	17.37	203

**Table 7:** The values of the equivalent circuit for the FeMnSi-(1.5-2)Cu alloy (HR samples).

R(QR)	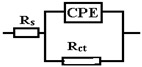	
Sample	FeMnSi-1.5Cu	FeMnSi-2Cu
R_s_ (ohm·cm^2^)	113	17
10^3^·Q (S·s^n^/cm^2^)	0.051	0.58
n	0.77	0.8
R_ct_ (ohm·cm^2^)	4013	138

## Data Availability

Data are contained within the article.
